# Polymorphisms in thymidylate synthase and reduced folate carrier (*SLC19A1*) genes predict survival outcome in advanced non-small cell lung cancer patients treated with pemetrexed-based chemotherapy

**DOI:** 10.3892/ol.2013.1175

**Published:** 2013-02-04

**Authors:** WEN-JUAN LI, HUA JIANG, XIN-JIAN FANG, HONG-LING YE, MING-HUAN LIU, YAN-WEN LIU, QIAN CHEN, LI ZHANG, JIN-YU ZHANG, CHUN-LUAN YUAN, QIU-YUN ZHANG

**Affiliations:** 1 Department of Medical Oncology, The Second People’s Hospital of Lianyungang (Lianyungang Hospital Affiliated to Bengbu Medical College), Jiangsu 222000, P.R. China

**Keywords:** pemetrexed, methylenetetrahydrofolate reductase, thymidylate synthase, *SLC19A1*, single nucleotide polymorphism, advanced non-small cell lung cancer

## Abstract

The aim of this study was to evaluate the association between thymidylate synthase (*TS*), methylenetetrahydrofolate reductase (*MTHFR*) and reduced folate carrier (*SLC19A1)* gene polymorphisms and the treatment efficacy of pemetrexed-based chemotherapy in advanced non-small cell lung cancer (NSCLC). Advanced NSCLC patients received pemetrexed and cisplatin every three weeks. Polymorphisms in the *TS*, *MTHFR* and *SLC19A1* genes were detected in peripheral blood samples using DNA sequencing and Taqman PCR. An analysis of gene polymorphisms was performed with respect to the progression-free survival (PFS), response rate (RR) and overall survival (OS) of patients treated with pemetrexed. The median PFS times for patients with the *TS* 2R/2R, 2R/3C or 3C/3C genotypes were significantly longer than those of patients with the 2R/3G, 3C/3G or 3G/3G genotypes (P=0.036). Patients with the *SLC19A1* CC genotype had a significantly longer median OS compared with individuals with the homozygous and heterozygous genotypes (12.2 vs. 8.9 and 7.3 months, respectively; P=0.022). The PFS and OS did not differ for the three genotypes of *MTHFR* assessed. The RR was higher in patients with the *TS* 2R/2R, 2R/3C or 3C/3C genotypes than in the other groups (P=0.044). The polymorphisms of the 5′-UTR of the *TS* gene and exon 6 (2522) C/T of the *SLC19A1* gene predict the survival of advanced NSCLC patients treated with pemetrexed. However, a large scale clinical trial is required to validate these findings.

## Introduction

Lung cancer is currently the most frequent cause of cancer mortality in the world and non-small-cell lung cancer (NSCLC) accounts for ∼80% of all lung cancer cases (1).Moreover, the 5-year survival of NSCLC is only 15% (2), as most patients are diagnosed at an advanced stage of the disease when surgery options are limited. Thus, chemotherapy has become the most important treatment option for treating these patients. Platinum-based chemotherapy is considered to be the foundation of treatment for patients with advanced NSCLC, with a median overall survival (OS) of 8–11 months, and therefore may prolong survival and improve the quality-of-life for these patients (3). However, current chemotherapeutic approaches have had limited success and therefore novel agents are urgently required to improve outcomes. Pemetrexed is a United States Food and Drug Administration (FDA)-approved therapy for advanced NSCLC (4) and a large phase III study has demonstrated that pemetrexed in combination with cisplatin provides similar efficacy and improved tolerability compared with gemcitabine in combination with cisplatin for advanced NSCLC patients (5). The study also indicated that pemetrexed provides similar improvements for patients with adenocarcinoma. Pemetrexed is widely used for first- and second-line and maintenance therapy in NSCLC (4–6). However, in China Pemetrexed is considered to be an expensive drug and is therefore not widely used. Consequently, it is critical to identify patients who may benefit from pemetrexed therapy in order to reduce the side-effects and provide the most cost-effective approach. A number of clinical trials have attempted to identify molecular biomarkers which may be associated with the response and toxicity of pemetrexed (7,8). The ultimate goal of this type of research is to create individualized treatment that provides an improved therapeutic profile by identifying the patients who are most likely to benefit from personalized therapy.

Pemetrexed is a multi-targeted, folate-based anti-metabolite that has been shown to have anticancer activity in other tumor types, including cervical (9), pancreatic (10), colorectal (11), breast (12) and gastric (13) cancers. Pemetrexed inhibits several enzymes in the thymidine and purine biosynthetic pathways, including thymidylate synthase (*TS*), dihydrofolate reductase (*DHFR*), glycinamide ribonucleotide formyl transferase (*GARFT*) and aminoimidazole carboxamide ribonucleotide-formyl transferase (*AICARFT*). Pemetrexed is transported into the cell by the reduced folate carrier (*RFC*) system and is then rapidly and extensively polyglutamated by folylpolyglutamate synthase (*FPGS*). The drug is eliminated from the cell by γ-glutamyl hydrolase (*GGH*).

*TS* is a folate-dependent enzyme and the presence of a single nucleotide polymorphism (SNP) in the *TS* promoter has been shown to be an independent factor in metastatic colorectal cancer patients treated with 5-fluorouracil (5-FU) (14). Methylenetetrahydrofolate reductase (*MTHFR*) is a key enzyme in the folate metabolism pathway and *MTHFR* polymorphisms have been reported to correlate with the outcome of patients treated with methotrexate (15) and pemetrexed (16,17). The reduced folate carrier *SLC19A1* is responsible for the transport of pemetrexed and therefore genetic variations in the gene may affect the transport of the drug (18). The associations between the gene polymorphisms and drug efficacy remain controversial. At present, few studies have investigated the correlation between pemetrexed pathway-associated gene polymophisms and drug efficacy. Therefore, the present study examined the association between the gene polymorphisms and therapeutic results of pemetrexed treatment in NSCLC patients.

## Patients and methods

### Patient selection

Chemotherapy-naive patients who were ≥18-years-old with histological or cytological evidence of measurable metastases or stage IIIB or IV NSCLC were enrolled in the present study. Other inclusion criteria included an Eastern Cooperative Oncology Group performance status (ECOG PS) ≤2 and normal organ function, including adequate hepatic, renal and hematological function. Patients who had brain metastases were eligible if they had a life expectancy ≥ three months. Pregnant or lactating women were excluded from the study. All patients provided written informed consent prior to the initiation of treatment. Separate informed consent was obtained for the collection of blood samples for the study of single-nucleotide polymorphisms (SNPs). This study was approved by the ethics committee of the Second People’s Hospital of Lianyungang (Jiangsu, China).

### Treatment

Pemetrexed (500 mg/m^2^) was administered to patients as a 10 min intravenous (i.v.) infusion on day 1 of a 21 day cycle with cisplatin (75 mg/m^2^, i.v.) administered on days 1–3 every three weeks. All patients received daily oral supplements of folic acid (350–1000 μg) 5–7 days prior to the first day of administration and the supplementation was continued for three weeks after the treatment ended. Vitamin B_12_ (1000 μg) was administered by intramuscular (i.m.) injection every three cycles of treatment. Dexamethasone (4 mg) was administered orally twice a day on the day before, day of and day after each dose of pemetrexed therapy to prevent allergic reactions.

### Treatment evaluation

All patients received complete clinical and laboratory evaluations prior to each cycle of treatment, including patient histories, physical examination, complete blood count, serum chemistry profile, electrocardiography (ECG) and radiological evaluation. Computed tomography (CT) and/or magnetic resonance imaging (MRI) were performed to evaluate the tumor response after every two cycles of therapy. The treatment effect was assessed according to the Response Evaluation Criteria in Solid Tumors (RECIST) (19) and categorized as complete response (CR), partial response (PR), stable disease (SD) or progressive disease (PD).

### DNA extraction

A 5 ml aliquot of blood was collected from each patient into an EDTA-coated tube before they underwent the first drug cycle. Genomic DNA was extracted from peripheral blood cells using the Blood DNA kit (Omega Bio-Tek, Norcross, GA, USA) according to the manufacturer’s instructions.

### TS genotyping

DNA was amplified by polymerase chain reaction (PCR) using the following primers: 5′-AACTGTGCTGCT GGCTTAGA-3′ (forward) and 5′-GTCTGTAAGGCGAG GAGGAC-3′ (reverse). The *TS* gene promoter repeat and SNP (two or three repeats; G>C within the second repeat of the 3R allele) polymorphisms were directly detected by DNA sequencing using an ABI Prism 3730 DNA analyzer (Applied Biosystems, Carlsbad, CA, USA). The *TS* genotype was divided into low expression genotypes (2R/2R, 2R/3C and 3C/3C) and high expression genotypes (2R/3G, C/3G and 3G/3G) according to the SNP and *TS* genotype.

### MTHFR and SLC19A1 SNP

*MTHFR* and *SLC19A1* SNPs were detected using a Taqman minor groove binder (MGB) probe-based assay. The PCRs for the *MTHFR* and *SLC19A1* genotypes were performed with a total volume of 5 μl, including 2.5 μl Taqman Master Mix, 0.25 μl Taqman MGB primer probe, 1 μl DNA and 1.25 μl DEPC H_2_O. The PCR cycling parameters were as follows: 45 cycles of 95°C for 10 min, followed by 92°C for 15 sec and 60°C for 90 sec. Genotyping was performed using the ABI 7900 HT Sequence Detector (Applied Biosystems) and analyzed by the Allelic Discrimination program (Applied Biosystems).

### Statistical analysis

SPSS version 13.0 (SPSS, Inc., Chicago, IL, USA) was used for the data analyses. The primary end point was progression-free survival (PFS) and the secondary end points were objective response rate (RR) by RECIST and OS. PFS was measured from the time of randomization to disease progression or mortality from any cause, whichever occurred earlier. OS was measured from the time of randomization to the time of mortality from any cause. The Hardy-Weinberg equilibrium for gene polymorphisms was used to compare the observed distributions of genotype frequencies. Fisher’s exact test was used to compare the treatment RRs between the genotypes. Kaplan-Meier curves and log-rank tests were also used to compare the OS and PFS distributions between the different SNP subgroups. P≤0.05 was considered to indicate a statistically significant difference and all values were two-sided.

## Results

### Characteristics

Between August 2009 and December 2011, a total of 47 patients were enrolled in the trial conducted at the Lianyungang hospital, which is affiliated with Bengbu Medical College. Two patients refused additional treatment after the first cycle administration due to grade 4 neutropenia. There were no significant differences in the baseline characteristics of the 45 patients and all the patients had adenocarcinoma. The median age was 63 years (range, 39–81 years). The characteristics of all the patients are shown in Table I.

### Genotypes

The gene information was obtained from the HapMap database. Table II shows the genotype distributions of *TS*, *MTHFR* and *SLC19A1*. The distributions of the genetic variants followed the Hardy-Weinberg equilibrium (P>0.05).

### Association between gene polymorphisms and the treatment response to pemetrexed

There were 14 patients in the study who achieved a PR, eight with PD, 22 with SD and one who achieved a CR. The overall response rate (ORR; CR + PR) was 33.3% (15/45). All 45 patients had evaluable data for analysis. No significant associations were observed between the response rate and polymorphisms of *MTHFR* and *SLC19A1*. Patients with 2R/2R, 2R/3C or 3C/3C genotypes had a significantly higher RR than patients with 2R/3G, 3C/3G or 3G/3G genotypes (53.3 vs. 23.3%, respectively; P=0.044; Table III).

### Survival analysis in patients with gene polymorphisms

**Figure 1:**
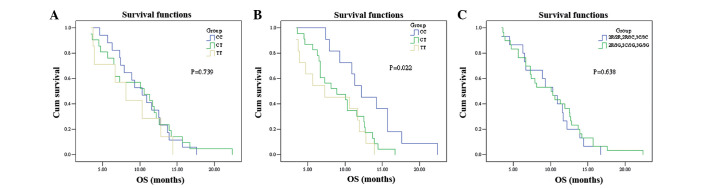
Kaplan-Meier curves estimating the association between the SNPs of the three genes and PFS of NSCLC patients treated with pemetrexed. (A) *MTHFR*, (B) *SLC19A1* and (C) *TS*. SNPs, single nucleotide polymorphisms; PFS, progression-free survival; NSCLC, non-small cell lung cancer; *TS*, thymidylate synthase; *MTHFR*, methylenetetrahydrofolate reductase.

**Figure 2:**
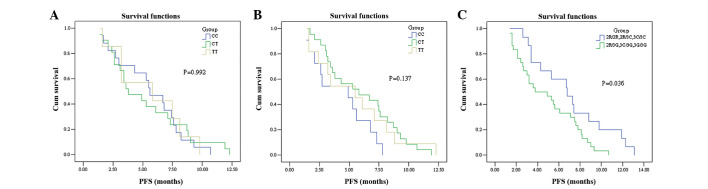
Kaplan-Meier curves estimating the association between the SNPs of the three genes and the OS of NSCLC patients treated with pemetrexed. (A) *MTHFR*, (B) *SLC19A1* and (C) *TS*. SNPs, single nucleotide polymorphisms; OS, overall survival; NSCLC, non-small cell lung cancer; *TS*, thymidylate synthase; *MTHFR*, methylenetetrahydrofolate reductase.

According to the Kaplan-Meier survival analysis, patients with the *TS* 2R/2R, 2R/3C or 3C/3C genotypes had a significantly longer median PFS than patients with the 2R/3G, 3C/3G or 3G/3G genotypes (6.8 vs. 3.8 months, respectively; log-rank x2 = 4.417; P=0.036; Fig. 1A). However, no significant difference in OS was observed between the two groups (P=0.638; Fig. 2A). The Kaplan-Meier survival analysis showed that PFS and OS did not differ for the three genotypes of *MTHFR* (PFS: CC, 5.6 months; CT, 3.8 months; TT, 5.8 months; log-rank x2 = 0.016; P=0.992; OS: 10.3 vs. 10.6 vs. 8.1 months, respectively; P=0.739; Fig. 1B and 2B). No significant association was observed between the PFS of patients with the *SLC19A1* CC genotype and patients with the CT and TT genotypes (log-rank x2 = 3.976; P=0.137; Fig. 1C). However, patients with the *SLC19A1* CC genotype had a longer OS compared with the other two groups (12.2 vs. 8.9 and 7.3 months, respectively; P=0.022; Fig. 2C). The PFS and OS summaries based on SNP analysis are shown in Table IV.

## Discussion

The present study was conducted to investigate the association of polymorphisms in pemetrexed-related genes with the clinical outcome in advanced NSCLC. The results suggest that the polymorphisms in the *TS* 5′-untranslated region (UTR) may result in differences in the PFS of NSCLC patients treated with pemetrexed-based chemotherapy. The response rate was higher in patients with the *TS* 2R/2R, 2R/3C or 3C/3C genotypes. Patients with the *SLC19A1* exon 6 (2522) CC genotype had improved OS when treated with pemetrexed compared with the patients without the genotype. However, a large-scale randomized prospective clinical trial is required to confirm these findings.

The *TS* gene is located on chromosome 18p11.32 and the enzyme encoded by it is essential for cell proliferation as it catalyzes the methylation of deoxyuridine-5′-monophosphate (dUMP) to form deoxythymidine-5′-monophophate (dTMP). It is known that *TS* is a potential target for fluoropyrimidine and other drugs, including capecitabine, raltitrexed and pemetrexed. The promoter enhancer region of the *TS* gene is prone to polymorphisms with double or triple repeats of 28-bp tandem repeats in the 5′-UTR. Mandola *et al* reported that the 3R alleles have a G>C SNP at the 12th position of the second repeat (20). Based on this SNP and the *TS* genotype, the 2R/3G, 3C/3G and 3G/3G genotypes were considered to be high expression types and the 2R/2R, 2R/3C and 3C/3C genotype were considered to be low expression types. However, no consensus was reached on the correlation between the *TS* polymorphisms and efficacy of 5-FU. Several studies have noted that a C SNP was associated with longer PFS in colorectal cancer patients treated with 5-FU (21,22). However, several other studies did not find any significant difference between *TS* SNPs and the clinical outcome of patients treated with 5-FU (23–25).

Few studies have investigated the association between *TS* genotypes and the efficacy of pemetrexed. Smit *et al* showed that there was no correlation between the high and low *TS* expression genotypes and the clinical outcome of advanced NSCLC patients treated with pemetrexed (16,17). In the present study, the PFS was significantly longer in patients with the 2R/2R, 2R/3C or 3C/3C genotypes compared with patients with the 2R/3G, 3C/3G or 3G/3G genotypes (P=0.036). However, no significant differences in OS were observed between the two groups (P=0.638). Further studies in larger populations are required to confirm these results, however, possible explanations include differences in the schedules of pemetrexed-based chemotherapy, types of lung cancer and ethnic populations.

*MTHFR* is a central regulatory enzyme involved in folate metabolism and the cellular concentrations of 5,10-methylenetetrahydrofolate are regulated by the activity of *MTHFR*(26). Moreover, optimal *TS* inhibition occurs when the cellular concentrations of 5,10-methylenetetrahydrofolate are elevated (27). The most common gene polymorphism linked with enzyme activity is *MTHFR*677 C/T (28). At present, the association between *MTHFR* SNPs and the efficacy of 5-FU is inconclusive and few studies have investigated the association of SNPs in this gene with the efficacy of pemetrexed. Jakobsen *et al* reported that the RR was significantly higher in patients with the *MTHFR* TT genotype compared with two other genotype groups in metastatic colorectal cancer patients treated with 5-FU (29). However, several large-scale clinical trials did not identify a clear association (30–32). Moreover, a large meta-analysis indicated that *MTHFR* 677C/T is not a reliable predictor of the efficacy of FU-based therapy (33). Smit *et al* reported that patients with an *MTHFR* TT homozygous mutation had increased PFS compared with wild-type or heterozygous NSCLC patients treated with pemetrexed [7.9 months, (95% CI, 3.9–16 months) vs. 2.9 months, (95% CI, 2.8–3.4 months); P=0.03] in (16). However, Argiris *et al* reported contrasting results (17). In the present study, no correlations were observed between the *MTHFR* genotypes and efficacy of pemetrexed in advanced adenocarcinoma lung cancer. It is possible that the present results were due to the small sample size of the study or due to only one SNP in *MTHFR* being investigated. This may have prevented the identification of possible pharmacogenetic associations, since drugs may exert their anticancer effects through multistep, multigenic cascades (30).

Pemetrexed is transported into cells by the RFC protein, which is encoded by the *SLC19A1* gene. In previous studies, the *SLC19A1* exon 6 (2522) CC genotype was observed to be associated with longer PFS and three-month progression-free status (progression-free at three months) in NSCLC patients treated with a combination of pemetrexed and bevacizumab chemotherapies (34). Adjei *et al* also demonstrated that a polymorphism in *SLC19A1* may predict survival differences in pemetrexed-treated NSCLC patients (18). In the present study, patients with the *SLC19A1* CC genotype had a longer median OS than patients with the other two genotypes (12.2 vs. 8.9 and 7.3 months, respectively; log-rank χ^2^ = 2.957; P=0.022). The present results are similar to those of the two previously described trials.

In summary, polymorphisms in the 5′-UTR of the *TS* gene and exon 6 (2522C/T) of the *SLC19A1* gene appear to be potential prognostic factors for NSCLC patients treated with pemetrexed. Notably, germline polymorphisms have been reported to correlate with response and/or toxicity. The ultimate aim of this research is to aid the identification of the patients who are most likely to benefit from pemetrexed-based chemotherapy. Therefore, SNPs may be a useful tool for creating optimal individualized treatment regimens.

## Figures and Tables

**Table I:** Clinical characteristics.

Characteristic	Data
Gender, n (5)	
Male	23 (51.1)
Female	22 (48.9)
Age (years)	
Median age	63
Range	39–81
ECOG, n (%)	
0	12 (26.7)
1	24 (53.3)
2	9 (20.0)
Stage of disease, n (%)	
IIIB	21 (46.7)
IV	24 (53.3)
Smoking status, n (%)	
Former/current smoker	31 (68.9)
Never-smoker	14 (31.1)
Number of cycles, n	
Total	199
Median	4
Range	1–6

ECOG, Eastern Cooperative Oncology Group.

**Table II:** Distribution of *TS*, *MTHFR* and *SLC19A1* genotypes.

Genotype	db SNP id	N (%)
*TS*	rs45445694	
2R/2R		0 (0)
2R/2C		7 (15.6)
3C/3C		8 (17.8)
2R/3G		12 (26.7)
3G/3G		4 (8.9)
3C/3G		14 (31.1)
*MTHFR* C677T	rs1801133	
CC		17 (37.8)
CT		21 (46.7)
TT		7 (15.6)
*SLC19A1* exon 6 (2522) C/T	rs1051298	
CC		11 (24.4)
CT		23 (51.1)
TT		11 (24.4)

SNP, single nucleotide polymorphism; *TS*, thymidylate synthase; *MTHFR*, methylenetetrahydrofolate reductase.

**Table III:** Response rate and gene polymorphisms.

Gene polymorphism	CR + PR	PD + SD	P-value
*TS*			0.044
2R/3G, 3C/3G, 3G/3G	7	23	
2R/2R, 2R/3C, 3C/3C	8	7	
*MTHFR*			0.306
CC	8	9	
CT	5	16	
TT	2	5	
*SLC19A1*			0.701
CC	3	8	
CT	9	14	
TT	3	8	

CR, complete response; PR, partial response; PD, progressive disease; *TS*, thymidylate synthase; *MTHFR*, methylenetetrahydrofolate reductase.

**Table IV:** PFS and OS according to SNPs.

Gene genotype	PFS (months)			OS (months)		
	Median	95% CI	P-value	Median	95% CI	P-value
*TS*			0.036			0.638
2R/2R, 2R/3C, 3C/3C	6.8	4.3–9.3		10.3	6.5–12.7	
2R/3G, 3C/3G, 3G/3G	3.8	1.0–6.6		10.1	7.8–12.8	
*MTHFR*			0.992			0.739
CC	5.6	3.7–7.5		10.3	7.6–13.0	
CT	3.8	1.6–6.0		10.6	4.6–16.6	
TT	5.8	0.0–12.5		8.1	4.5–11.7	
*SLC19A1*			0.137			0.022
CC	4.9	2.0–7.8		12.2	8.6–15.8	
CT	5.8	2.0–9.6		8.9	4.5–13.3	
TT	5.5	2.4–8.6		7.3	0.9–13.7	

PFS, progression-free survival; OS, overall survival; SNP, single nucleotide polymorphism; *TS*, thymidylate synthase; *MTHFR*, methylenetetrahydrofolate reductase.
